# Dosimetric benefits of 1.5 T MR-guided radiotherapy in partial breast treatment

**DOI:** 10.1371/journal.pone.0342973

**Published:** 2026-03-12

**Authors:** Jiwon Sung, Gyuri Song, Inho Jeong, Jinsook Ha, Yeona Cho, Joongyo Lee, Jihun Kim

**Affiliations:** 1 Department of Radiation Oncology, Gangnam Severance Hospital, Yonsei University College of Medicine, Seoul, South Korea; 2 Department of Radiation Oncology, Gil Medical Center, Gachon University College of Medicine, Incheon, South Korea; Chung-Ang University Gwangmyeong Hospital, KOREA, REPUBLIC OF

## Abstract

This study aimed to evaluate the dosimetric benefits of magnetic resonance (MR)-guided radiotherapy (MRgRT) compared to computed tomography (CT)-guided RT for partial breast treatment. First, treatment plan quality was compared between MRgRT with step-and-shoot (S&S) IMRT and CT-guided RT with volumetric-modulated arc therapy (VMAT). Second, the dosimetric benefit of online adaptation in MRgRT was assessed by comparing the initial planned doses with daily MR-defined target geometry to those with planning CT-defined target geometry. Plan quality for MRgRT was comparable to, or slightly lower than, CT-guided RT. The V_95%_ of the planning target volume (PTV) was lower by 1.72% and 1.94%, respectively, with a lower minimum dose and a higher maximum dose. For the OARs, the dosimetric parameters were generally similar, but for the skin, the D_max_, D_0.5mL_, and D_1mL_ were slightly higher in MRgRT. Evaluation of the initial planned doses with the daily MR-based target definition resulted in a maximum deviation of 46% for the PTV V_95%_, demonstrating uncertainty in CT-based target definition. Although the MRgRT planning quality may be slightly compromised due to the S&S IMRT technique, the dosimetric benefits from online adaptation can be substantial when CT-based target definitions need to be modified based on daily MR imaging guidance.

## 1. Introduction

Partial breast irradiation (PBI) has been increasingly utilized in postoperative breast radiotherapy for patients with early-stage breast cancer [1,2]. This treatment has shown comparable efficacy to whole breast irradiation consisting of 5 weeks of external beam radiotherapy with or without an additional 1–2 weeks of boost irradiation [3,4], with superior outcomes in terms of acute and late treatment-related toxicity and cosmesis [5,6]. As radiation beams in PBI are targeted only at a part of the breast volume, accurately identifying the target volume is essential. While PBI target identification is typically achieved by locating surgical clips inserted during breast surgery or detecting a seroma, challenges arise because of potential clip migration or variations in seroma volume throughout radiation treatment [7–9].

Magnetic resonance (MR)-guided RT (MRgRT) can be an optimal treatment technique for PBI because of high soft tissue contrast during daily MR imaging, potentially leading to accurate target definition [10]. In MRgRT, daily MR images are acquired before treatment beam irradiation, whereas in conventional RT, cone-beam computed tomography (CT) or megavoltage CT images are obtained. Detailed visualization of soft tissues, particularly seroma, is a notable advantage of MR imaging [11–13]. When changes in anatomic shape or seroma volume are detected in daily MR images, MRgRT can modify the initial structure based on the image findings and can establish online adaptive plans accordingly. Because of these advantages, recent studies have focused on determining the appropriate planning target volume (PTV) margin size and evaluating the PTV margin in MR-Linac systems [14–16]. For PBI, reducing the PTV margin to 3 mm or less in MRgRT using 0.35 T MR-Linac may offer better normal tissue protection than that with volumetric-modulated arc therapy (VMAT) with a 10 mm margin [17].

However, treatment machines currently used for MRgRT have some limitations when compared to conventional Linacs. The Elekta Unity (Elekta AB, Stockholm, Sweden) and ViewRay MRIdian (ViewRay, Inc, Oakwood Village, OH), which are currently available commercial MR-Linacs, can only conduct step-and-shoot (S&S) intensity-modulated radiation therapy (IMRT). Because of this limitation, the resulting treatment plan quality of S&S IMRT using MR-Linac may be lower than that of VMAT using conventional Linacs. A comparative study of treatment plans for patients with prostate and rectal cancer showed a decrease in plan homogeneity and increase in the PTV D_mean_, D_1%_, and doses to organs at risk (OARs) with Elekta Unity’s S&S compared to the conventional Linac’s VMAT [18]. In cases of anal cancer, while doses for target and OARs were comparable, the PTV_45Gy_ D_98%_ coverage was compromised in 3 out of 10 MR-Linac plans [19]. For APBI treatment, the ViewRay MRIdian’s S&S IMRT results were similar to or slightly worse than those of VMAT [17]. However, for PBI using MR-Linac, plan quality evaluations have only been conducted for ViewRay MRIdian, with no studies addressing the dosimetric benefits related to the changes in target position and shape during treatment.

Therefore, this study aimed to comprehensively evaluate the dosimetric benefits of MRgRT over CT-guided RT for partial breast radiotherapy. First, the plan quality of S&S IMRT plans generated on the MRgRT system and VMAT plans created on a conventional linac was compared using the same CT images and identical contour structures. In the second, the MR-based target structures modified at the treatment were applied to the dose distribution of the original CT-based MRgRT initial plan. This enabled a quantitative assessment of the dose differences that would occur if each patient were treated with the initial non-adapted plan, thereby allowing an evaluation of the dosimetric advantages of online adaptation.

## 2. Materials and methods

### 2.1. Patient data

This study was a retrospective analysis approved by the Institutional Review Board (IRB) of the Yonsei University Health System (IRB No. 3-2024-0233). The study was conducted through a retrospective review of medical records, and access to the data for research purposes was granted on August 14, 2024. All patient data were fully anonymized during and after data collection, and the investigators had no access to any information that could directly identify individual participants. The study included 20 breast cancer patients who underwent MRgRT using the adaptive-to-shape (ATS) workflow on an Elekta Unity system [20]. For all patients, the PTV was adjusted through the ATS process based on the visibility of the tumor bed on the daily MR image acquired on the first treatment day. Table 1 shows patient clinical information as well as the differences between the initial PTV defined on the planning CT and the modified PTV revised based on the daily MR image acquired at the first treatment fraction.

**Table 1:** Patient characteristics.

#	Stage	Prescribed dose (Gy)	Aim	Location	Initial PTV (mL)	1^st^ fraction modified PTV (mL)	Volume difference (mL)	Difference in centroid position (mm)
1	pT1aN0M0	30	PBI	Right	37.9	12.2	−25.7	6.0
2	pTisNxM0	30	PBI	Right	57.0	67.8	10.8	8.5
3	pTisN0M0	30	PBI	Left	258.5	273.7	15.2	6.3
4	pT1cN0M0	30	PBI	Left	109.9	134.5	24.6	7.2
5	pT1cN0M0	30	PBI	Right	80.7	89.9	9.2	5.2
6	pTisN0M0	30	PBI	Left	100.1	109.1	9.0	3.1
7	pT1cN0M0	30	PBI	Left	87.5	90.3	2.8	3.0
8	pT1cN0M0	30	PBI	Right	46.4	47.9	1.5	4.4
9	pT1cN0M0	30	PBI	Right	39.2	45.9	6.7	3.7
10	pT1bN0M0	30	PBI	Left	73.3	72.6	−0.6	2.3
11	Phyllodes tumor	10	Boost	Right	63.8	65.7	1.9	5.2
12	ypT0NmiM0	10	Boost	Right	142.8	93.2	−49.6	22.6
13	pT2N0M0	10	Boost	Right	68.1	12.8	−55.3	9.1
14	pT2N0M0	10	Boost	Right	32.1	8.1	−24.0	8.9
15	pT1bN1M0	10	Boost	Left	62.8	33.4	−29.4	1.7
16	pT1aN1M0	10	Boost	Right	24.0	46.6	22.6	9.6
17	pT2N0M0	10	Boost	Left	24.1	31.0	6.9	11.6
18	pTisN0M0	10	Boost	Left	68.2	75.6	7.4	3.5
19	pT1cN0M0	10	Boost	Right	27.0	32.8	5.8	5.6
20	pTisN0M0	10	Boost	Left	40.5	43.4	2.9	8.3

“-” indicates a reduction in the PTV volume compared to the initial PTV volume.

To evaluate the dosimetric benefit of online adaptation in MRgRT associated with PTV changes, the 20 patients were classified into three groups according to patterns of PTV positional and volumetric changes based on the results shown in Table 1. First, three patients (Patients 12, 16, and 17) who showed prominent positional changes between the initial PTV and the modified PTV in both visual assessment and quantitative centroid differences were separately classified as Group 1 (positional-change group). Group 2 (volumetric-change group) consisted of seven patients whose absolute PTV volume change exceeded 10 mL. Finally, Group 3 (minimal-change group) included the remaining ten patients who showed relatively small changes in both PTV volume and position.

### 2.2. Acquisition of planning CT and delineation of structures

**Fig 1:**
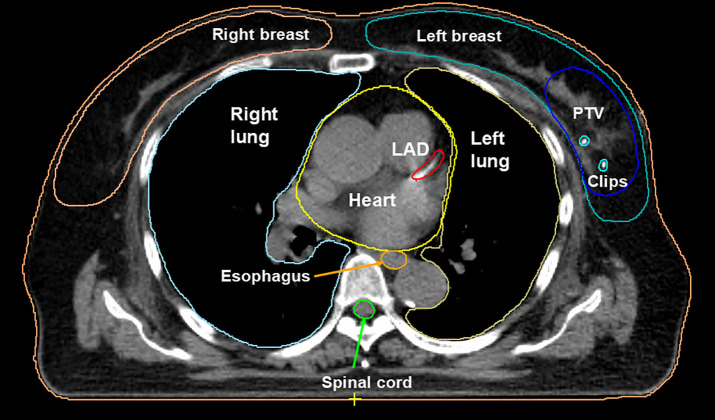
Axial views of the PTV and OARs contoured using the planning CT image. PTV, planning target volume; OARS, organs at risk.

Planning CT images were acquired in the supine position using a head cushion, wing board, and knee step provided by Elekta, with a 2 mm slice thickness. Target volumes were defined based on the planning CT image according to the departmental standard protocols using MIM Maestro v7.2 (MIM Software Inc., Cleveland, OH). Initially, the tumor bed was defined as the surgical cavity, including surgical clips and any changes in the surrounding tissue architecture. A 1-cm margin around the tumor bed was used to create the clinical target volume (CTV). Subsequently, it was modified to exclude the chest wall and a 5-mm strip of skin [21]. As in cases where the PTV was set equal to the CTV during PBI treatment with CyberKnife [22], the PTV was defined the same as the CTV in this study because MRgRT allows for robust visualization of the tumor bed, target modification in every treatment, and motion monitoring to detect intrafractional variation. OARs (ipsilateral breast, contralateral breast, lung, heart, and skin) were also contoured according to standard departmental guidelines (Fig 1). All structures were contoured and verified by the board-certified radiation oncologist responsible for each patient.

### 2.3. Treatment planning

**Fig 2:**
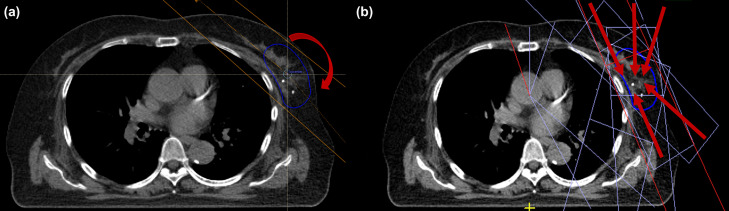
Examples of the (a) VMAT and (b) S&S IMRT plan setup and beam layout on the planning CT image. The number of red arrows indicates the number of beams, and the red arrow direction indicates beam direction (Blue: PTV).

VMAT plans for Versa HD (Elekta AB, Stockholm, Sweden) were created with the beam isocenter set at the center of the target. To facilitate a fair comparison between the MRgRT and CT-guided RT plans, all the treatment plans were generated to ensure that both plans for the same patient were of the highest possible quality and consistency, following the departmental dosimetric criteria (Table 2). Since each patient had a different target size or location, optimization objectives were individually fine-tuned to minimize OAR doses while maintaining PTV dose constraints. Single-arc VMAT plans for the CT-guided RT were generated with gantry angle intervals of approximately 180–200°, with a minimum segment of 5 mm at the isocenter for delivery on Versa HD (Fig 2(a)). All plans were calculated using a collapsed cone convolution algorithm with a 3 mm calculation grid spacing in RayStation v5.0.3 (RaySearch Lab, Stockholm, Sweden).

**Table 2:** Summary of the dosimetric criteria used for optimization of all S&S IMRT and VMAT plans as per local departmental protocols and evaluation parameters for plan comparison.

Structure	Dosimetric criteria	Evaluation parameters
**PTV**	D_95%_ ≥ 28.5 Gy (PBI) D_95%_ ≥ 9.5 Gy (Boost)	V_95%_, D_max_, D_min_, HI, CI
	D_max_ < 107% (PBI) D_max_ ≤ 110% (Boost)	
**Ipsilateral breast**	–	V_100%_, V_50%_
**Contralateral breast**	D_mean_ ≤ 2 Gy (PBI) D_mean_ ≤ 0.5 Gy (Boost)	D_max_
**Ipsilateral lung**	–	D_mean_, V_5Gy_, V_10Gy_ (PBI), V_17Gy_(PBI)
**Heart**	D_mean_ ≤ 1 Gy (PBI) D_mean_ ≤ 0.5 Gy (Boost)	D_mean_
**Skin**	–	D_max_, D_0.5mL_, D_1mL_

MRgRT plans were generated using Monaco 5.51 (Elekta AB, Stockholm, Sweden). The S&S IMRT plans were generated using 4–6 beams and fewer than 150 segments with 7 MV flattened filter-free beams (Fig 2(b)). Optimization objectives were adjusted for individual patient plans to achieve the departmental dosimetric criteria summarized in Table 2. All plans were calculated using a GPU-accelerated Monte Carlo dose calculation algorithm with 1% dose uncertainty and a 3 mm calculation grid spacing. Plans were considered acceptable when all objectives were met.

### 2.4. Comparison of plan quality: S&S IMRT vs. VMAT

Using the contoured structures based on the planning CT, the S&S IMRT plans for MRgRT were dosimetrically compared to VMAT plans retrospectively generated for CT-guided RT using Elekta Versa HD. All plans were compared using the evaluation parameters shown in Table 2. The conformity index (CI) and homogeneity index (HI) were calculated using the following formulas: [23]


$$CI=\frac{Total\;body\;V_{95\%}}{PTV}$$

where total body V_95%_ represents the volume receiving 95% of the prescribed dose in the patient body.


$$HI=\frac{D_{5\%}}{D_{95\%}}$$

where D_5%_ and D_95%_ represent the minimum doses to 5% and 95% of the PTV, respectively. Statistical analysis for all measures used the Student’s t-test, with statistical significance set at values of p < 0.05.

### 2.5. Evaluation of dosimetric benefits of online adaptation in MRgRT

**Fig 3:**
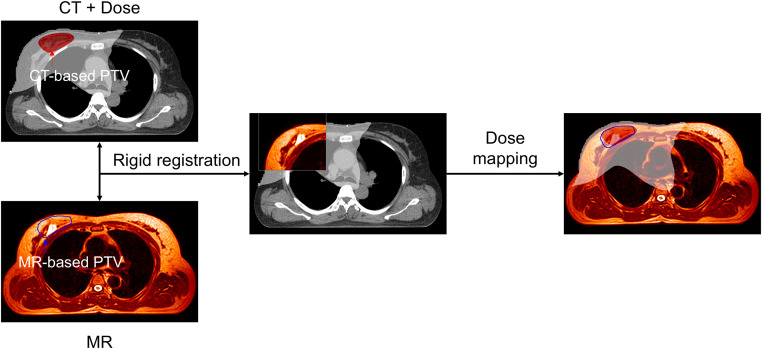
Schematic illustration of the dose mapping workflow for Patient 10. The CT-based dose distribution was rigidly registered to the daily MR image acquired at the first treatment fraction and mapped onto the MR-based recontoured PTV. Red and white isodose lines represent 95% and 10% of the prescribed dose, respectively. The CT-based PTV is shown in red, and the MR-based recontoured PTV is shown in blue.

To evaluate the dosimetric benefits of online adaptation in MRgRT, we analyzed the impact of uncertainties in CT-based target definition. This analysis was designed to simulate a clinical scenario in which treatment is delivered without MR-guided adaptation, relying solely on CT-defined target structures. For each of the 20 patients, a conventional CT-based treatment plan was created using the RayStation treatment planning system for delivery on a Versa HD linear accelerator. The initial dose distribution from this plan was then mapped to the modified PTV structure, which was delineated on the MR image acquired on the first day of treatment (Fig 3). The PTV location and shape were redefined by board-certified radiation oncologists based on daily MR imaging during the online adaptation process. Rigid registration between the CT and MR images was performed using the sternum and the breast on the treatment side as anatomical landmarks, following standard CT and CBCT alignment protocols commonly used in conventional radiotherapy. Several dose volume histogram metrics for the PTV, including the volume receiving at least 95 percent of the prescribed dose (V_95_), the maximum dose (D_max_), the minimum dose (D_min_), the homogeneity index (HI), and the conformity index (CI), were calculated based on the modified PTV structure and compared with the corresponding values from the initial PTV in the reference plan.

## 3. Results

**Fig 4:**
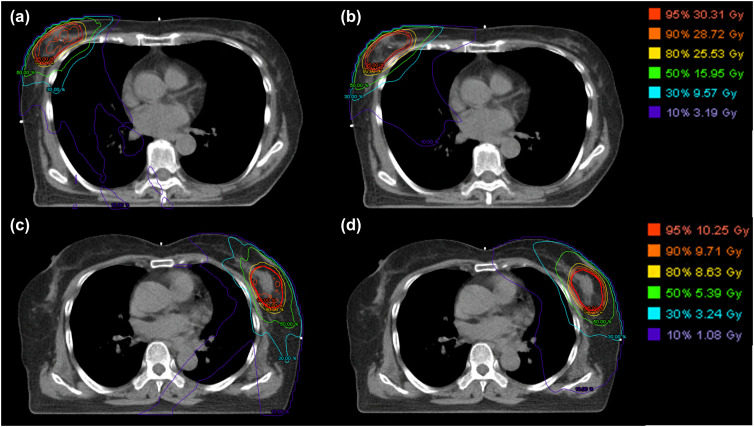
Comparison of dose distributions between the MR-guided S&S IMRT and CT-guided VMAT plans. **(a)** MR-guided S&S IMRT plan for the 30 Gy PBI treatment of Patient 1. **(b)** CT-guided VMAT plan for the 30 Gy PBI treatment of Patient 1. **(c)** MR-guided S&S IMRT plan for the 10 Gy boost treatment of Patient 18. **(d)** CT-guided VMAT plan for the 10 Gy boost treatment of Patient 18.

Clinically acceptable treatment plans were achieved for all the patients with MRgRT and CT-guided RT. Fig 4 compares the qualitative dose distributions of Patient 1 and 18, who received 30 Gy and 10 Gy, respectively, and Table 3 provides a quantitative analysis across all 20 patients. For the PTV coverage and uniformity, the VMAT plans of CT-guided RT showed slightly better results compared to the S&S IMRT plan of the MRgRT for both PBI and the boost treatment. The V_95%_ of MRgRT were 1.72% and 1.94% lower than those of CT-guided RT for PBI and the boost treatment, respectively (p = 0.003*, 0.002*). The D_min_ of the MRgRT were 7.89% and 1.27% lower than those of CT-guided RT, whereas the D_max_ of MRgRT were 0.83% and 5.31% higher than those of CT-guided RT for the PBI and boost treatment, respectively. Overall, the HI and CI of the MRgRT plans were similar or slightly worse compared to the CT-guided RT plans. For OARs, the dosimetric evaluation parameters of MRgRT are similar to those of CT-guided RT, except for the skin receiving PBI treatment. The D_max_, D_0.5mL_ and D_1mL_ to the skin were 3.6, 3.4, and 3.2 Gy higher in MRgRT than in CT-guided RT, respectively (p < 0.001*, p = 0.004*, 0.006*, respectively).

**Table 3:** Dosimetric comparison between the MR-guided IMRT and CT-guided VMAT plans for PBI and the boost treatment. All values are presented as the mean ± standard deviation across the 20 patients.

Structures	Parameters	PBI			Boost		
		MRgRT (S&S IMRT)	CT-guided RT (VMAT)	P-value	MRgRT (S&S IMRT)	CT-guided RT (VMAT)	P-value
PTV	V_95%_	97.92 ±1.51%	99.64 ±0.50%	0.003*	97.75 ±1.67%	99.69 ±0.20%	0.002*
	D_max_	106.00 ±1.33%	105.17 ±0.73%	0.101	109.70 ±2.36%	104.39 ±0.28%	p < 0.00001*
	D_min_	82.77 ±7.97%	90.66 ±3.32%	0.010*	83.78 ±7.96%	85.05 ±15.12%	0.817
	HI	1.06 ±0.02	1.05 ±0.01	0.089	1.09 ±0.03	1.04 ±0.01	p < 0.001*
	CI	1.29 ±0.14	1.20 ±0.07	0.061	1.19 ±0.12	1.23 ±0.07	0.439
Ipsilateral breast	V_100%_	11.60 ±6.29%	13.23 ±5.57%	0.549	7.25 ±3.79%	7.46 ±3.56%	0.901
	V_50%_	36.59 ±11.09%	36.87 ±10.40%	0.954	21.27 ±7.61%	22.49 ±8.89%	0.745
Contralateral breast	D_max_	4.32 ±4.70 Gy	3.91 ±3.25 Gy	0.821	1.14 ±0.67 Gy	0.77 ±0.65 Gy	0.228
Ipsilateral lung	D_mean_	1.65 ±0.61 Gy	1.97 ±0.43 Gy	0.193	0.69 ±0.26 Gy	0.70 ±0.27 Gy	0.987
	V_5Gy_	10.21 ±5.02%	10.89 ±3.58%	0.732	0.40 ±0.55%	0.20 ±0.40%	0.367
	V_10Gy_	2.13 ±1.11%	2.42 ±1.23%	0.591	–	–	–
	V_17Gy_	0.28 ±0.27%	0.17 ±0.12%	0.249	–	–	–
Heart	D_mean_	0.36 ±0.11 Gy	0.56 ±0.06 Gy	p < 0.0001*	0.38 ±0.25 Gy	0.33 ±0.11 Gy	0.616
Skin	D_max_	29.56 ±1.60 Gy	26.04 ±2.04 Gy	p < 0.001*	9.02 ±1.57 Gy	8.96 ±0.80 Gy	0.908
	D_0.5mL_	27.02 ±2.31 Gy	23.63 ±2.22 Gy	0.004*	7.48 ±1.68 Gy	7.67 ±1.19 Gy	0.774
	D_1mL_	26.18 ±2.38 Gy	22.95 ±2.24 Gy	0.006*	6.96 ±1.66 Gy	7.13 ±1.32 Gy	0.800

* Statistically significant difference (p < 0.05).

We simulated how much PTV doses changed compared to the initial planned doses during the treatment period. As described in Section 2.1 (Patient data), patients in this study were classified into three groups based on the differences between the CT-based PTV and the MR-based PTV modified using the daily MR image acquired at the first treatment fraction: Group 1, which exhibited pronounced positional changes; Group 2, which showed substantial volumetric changes; and Group 3, which demonstrated relatively minimal changes in both position and volume. Table 4 represents the results for Group 2 and 3 to evaluate the dose delivered to the PTV according to changes in volume. V_95%_, D_max_, D_min_, HI, and CI of the PTV that decreased more than 10 mL were similar to those of the initial PTV except for D_min_ and CI which increased by 20.14% and 2.95. For the PTV that increased more than 10 mL than the initial PTV, the V_95%_ and D_min_ were reduced by 16.72% and 71.52%, respectively. HI increased by 3.74 while the remaining dosimetric parameters were similar to those of the initial target. For PTVs whose changed volume ranged from −10 mL to 10 mL, the V_95%_ and D_min_ were reduced by 12.25% and 42.49%, respectively, while the HI increased by 0.25.

**Table 4:** Comparison of dosimetric parameters between the modified and initial PTV structures applied to the initial planned dose distributions for patients in Group 2 (∆V < −10 mL or ∆V > 10 mL) and Group 3 (−10 mL < ∆V < 10 mL).

Dosimetric parameters	∆V < −10 mL (n = 3)			−10 mL < ∆V < 10 mL (n = 10)			∆V > 10 mL (n = 4)		
	Modified PTV	Initial PTV	p-value	Modified PTV	Initial PTV	p-value	Modified PTV	Initial PTV	p-value
V_95%_	99.95 ±0.10%	99.27 ±0.67%	0.089	87.50 ±6.37%	99.75 ±0.18%	p < 0.00001*	83.16 ±5.30%	99.88 ±0.12%	0.005*
D_max_	103.76 ±0.63%	104.60 ±0.39%	0.064	104.48 ±0.60%	104.87 ±0.70%	0.195	104.61 ±0.82%	105.13 ±1.05%	0.534
D_min_	95.61 ±2.81%	75.47 ±21.68%	0.115	48.81 ±23.00%	91.30 ± 2.09%	0.005*	19.64 ±23.90%	91.16 ±4.81%	0.007*
HI	1.03 ±0.01	1.05 ±0.01	0.025*	1.30 ±0.25	1.05 ±0.01	p < 0.0001*	4.78 ±5.74	1.04 ±0.01	0.323
CI	4.14 ±1.69	1.19 ±0.04	0.013*	1.09 ±0.06	1.21 ±0.03	p < 0.0001*	1.00 ±0.06	1.19 ±0.12	0.067

‘**∆**’ indicates the difference in volume between the initial target structure and the modified target structure, where ‘-’ signifies a decrease. (n indicates the number of patients)

**Fig 5:**
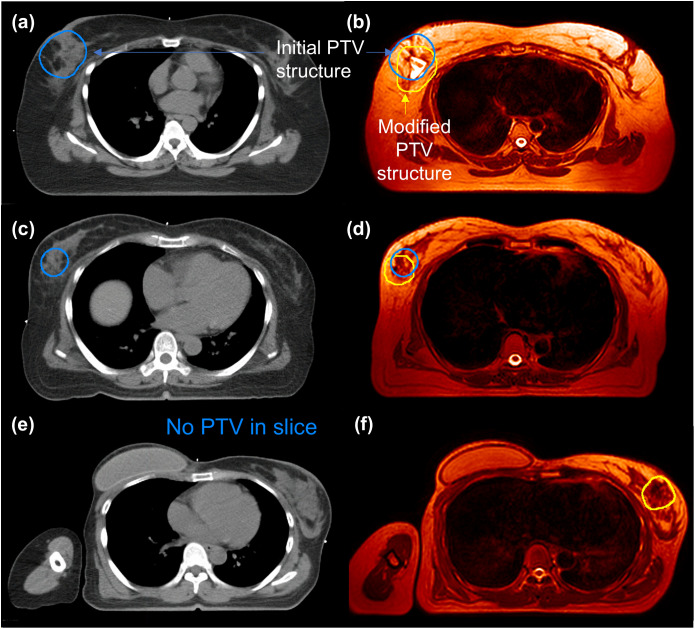
CT and MR images with PTV structures for Group 1 (Patients 12, 16, and 17) who showed changes in both the position and volume of the PTV compared to the initial structure. For Patient 12: reference CT **(a)**, daily MR **(b)**, and corresponding PTV structures. For Patient 16: reference CT **(c)**, daily MR **(d)**, and corresponding PTV structures. For Patient 17: reference CT **(e)**, daily MR **(f)**; no PTV is visible in the CT slice shown. Blue contours are the initial PTV structures based on reference CT, and yellow contours are the modified PTV structures based on daily MR.

As shown in Fig 5, Group 1 (Patient 12, 16, and 17) was noticeably altered in the daily MR images, substantial reductions in target coverage were observed when the initial CT-based dose distribution was mapped onto the modified PTVs. As shown in Table 5, while D_max_ remained comparable between the initial and modified PTVs, the coverage of the target volume was significantly reduced. For Patient 12, the V_95%_ and D_min_ of the modified PTV dropped to 53.3% and 23.6%, respectively, compared to 99.4% and 88.0% in the original CT-based PTV. Similar trends were observed in Patients 16 and 17. The V_95%_ decreased from 100.0% and 99.7% to 55.1% and 59.8%, respectively, and the D_min_ decreased from 94.9% and 85.9% to 16.3% and 3.8%, respectively.

**Table 5:** Comparison of dosimetric parameters between the modified PTVs based on daily MR images and the initial PTVs defined on reference CT images for Group 1. The initial dose distribution from the CT-based plan was mapped onto the daily MR-based PTVs to estimate the delivered dose.

Patient	Dosimetric parameters	Daily MR-based PTV (mapped with initial dose distribution)	Reference CT-based PTV (from initial dose distribution)
12	V_95%_ (%)	53.3	99.4
	Maximum dose (%)	103.3	103.9
	Minimum dose (%)	23.6	88.0
16	V_95%_ (%)	55.1	100
	Maximum dose (%)	104.0	104.4
	Minimum dose (%)	16.3	94.9
17	V_95%_ (%)	59.8	99.7
	Maximum dose (%)	104.4	104.8
	Minimum dose (%)	3.8	85.9

PTV, planning target volume; V_95%_, volume receiving 95% of the prescribed dose; D_max_, the maximum doses to the PTV; D_min_, the minimum doses to the PTV

## 4. Discussion

MRgRT enables the delivery of online adaptive radiotherapy based on daily MR imaging and is therefore considered suitable for PBI. Compared with CT imaging, MR imaging provides superior soft-tissue contrast, allowing clearer visualization of the surgical cavity and seroma. In addition, anatomical variations such as changes in seroma that may occur between CT simulation and the actual treatment course can be identified on daily MR images and addressed through online adaptation, representing an important advantage in PBI treatment. However, to the best of the authors’ knowledge, the dosimetric benefit of MRgRT-based online adaptation has not yet been evaluated for PBI. Therefore, this study aimed to assess the dosimetric advantages provided by MRgRT-based online adaptation in patients undergoing PBI.

First, plan quality between S&S IMRT and VMAT was compared to assess whether MRgRT is disadvantaged by limitations of the planning technique. The two techniques showed generally comparable plan quality with no clinically meaningful differences, consistent with previous studies [24–26]. Subsequently, this study evaluated the impact of image-based target definition on dosimetric outcomes. The PTV recontoured on the daily MR image acquired on the first treatment day was assumed to best represent the actual target shape and position. The dose delivered to this modified PTV was quantitatively evaluated by applying the same CT-based dose distribution from the initial treatment plan and compared with the dose to the original CT-based PTV in that plan.

We used only the first fraction MR based PTV because previous studies have shown that the largest anatomical changes occur between CT simulation and the start of treatment, while changes during later fractions are minimal [27,28]. In addition, both the PBI and boost treatments in this study consisted of five fractions delivered consecutively over a short treatment course, and meaningful anatomical changes were rarely observed after the first fraction.

Among the 20 patients, excluding the 3 with the most significant changes, 4 of the remaining 17 patients experienced a reduction in target volume by more than 10 mL, resulting in increased target coverage. However, for the remaining 13 patients, the PTV was mostly expanded, which included areas that were not initially covered by the original PTV, leading to a decrease in target coverage and minimum dose.

In the three cases, not only the shape and volume but also the position of the PTV showed changes. As shown in Table 1, the differences in the center positions between the CT-based initial PTV and the PTV redefined on the first-day MR image were 22.6 mm for Patient 12, 9.6 mm for Patient 16, and 11.6 mm for Patient 17. These positional differences can be explained by two factors. First, the actual target location may have changed due to seroma changes during the interval between CT simulation and the first treatment day. Second, the superior soft-tissue contrast of MR imaging allows the surgical cavity to be visualized much more clearly than on CT, enabling a more accurate redefinition of the PTV. As shown in Fig 5, Patient 12 (Fig 5(a), (b)) appears to exhibit a positional difference due to seroma change and the unclear seroma boundary on CT. For patients 16 and 17 (Fig 5C–(f)), the surgical cavity was visualized much more clearly on MR than on CT, allowing the postoperative structures to be identified more accurately and the PTV to be redefined accordingly.

When comparing the initial planned doses to the initial PTV with those to the modified PTV obtained after the online adaptive planning process, V_95%_ decreased by 46.1%, 45.0%, and 40.0%, respectively. While conventional linac systems typically use CBCT to acquire images for daily anatomical positioning, the lower resolution of CBCT makes it difficult to confirm the PTV’s position. In such situations, MRgRT can precisely identify the seroma and tumor bed positions using MR images and perform contour adjustments and replanning through online adaptive processes, making it specialized for partial breast treatment.

This study has several limitations. First, the dose distribution was not recalculated on the MR images. Instead, the dose from the CT-based treatment plan was transferred to the MR-defined structures using rigid registration. Therefore, the resulting dose distribution does not represent a physically accurate distribution based on the actual MR anatomy, but should be interpreted as a conceptual dosimetric estimation. Second, this study focused on evaluating PTV dose differences caused by visualization discrepancies between CT and MR images. As such, dose evaluations for OARs and cumulative anatomical changes over the treatment course were not included in the analysis. Therefore, we are planning a study that will incorporate physically accurate dose recalculation on MR images, deformable image registration, and quantitative dose evaluation for OARs.

## 5. Conclusion

While MRgRT using S&S IMRT may compromise plan quality compared to CT-guided RT using VMAT, it offers substantial advantages by allowing precise adjustments based on daily MR imaging. This capability to accurately visualize and adapt to changes in the target’s position and anatomy throughout the treatment course significantly enhances treatment accuracy and efficacy.
